# Local Entropy Optimization–Adaptive Demodulation Reassignment Transform for Advanced Analysis of Non-Stationary Mechanical Signals

**DOI:** 10.3390/e27070660

**Published:** 2025-06-20

**Authors:** Yuli Niu, Zhongchao Liang, Hengshan Wu, Jianxin Tan, Tianyang Wang, Fulei Chu

**Affiliations:** 1 Department of Mechanical Engineering, Tsinghua University, Beijing 100084, China; yuliniu0614@mails.tsinghua.edu.cn (Y.N.); liangzc20@mails.tsinghua.edu.cn (Z.L.); tanjianxin@suntien.com (J.T.); chufl@mail.tsinghua.edu.cn (F.C.); 2 School of Mechanical, Electronic and Control Engineering, Beijing Jiaotong University, Beijing 100091, China; 21121251@bjtu.edu.cn; 3 China Suntien Green Energy Co., Ltd., Shijiazhuang 050051, China

**Keywords:** time–frequency analysis, Rényi entropy, non-stationary signals, mechanical vibration signals, fault diagnosis

## Abstract

This research proposes a new method for time–frequency analysis, termed the Local Entropy Optimization–Adaptive Demodulation Reassignment Transform (LEOADRT), which is specifically designed to efficiently analyze complex, non-stationary mechanical vibration signals that exhibit multiple instantaneous frequencies or where the instantaneous frequency ridges are in close proximity to each other. The method introduces a demodulation term to account for the signal’s dynamic behavior over time, converting each component into a stationary signal. Based on the local optimal theory of Rényi entropy, the demodulation parameters are precisely determined to optimize the time–frequency analysis. Then, the energy redistribution of the ridges already generated in the time–frequency map is performed using the maximum local energy criterion, significantly improving time–frequency resolution. Experimental results demonstrate that the performance of the LEOADRT algorithm is superior to existing methods such as SBCT, EMCT, VSLCT, and GLCT, especially in processing complex non-stationary signals with non-proportionality and closely spaced frequency intervals. This method provides strong support for mechanical fault diagnosis, condition monitoring, and predictive maintenance, making it particularly suitable for real-time analysis of multi-component and cross-frequency signals.

## 1. Introduction

The signals generated by rotating machinery under complex operating conditions often exhibit nonlinear, time-varying, and multi-component characteristics, which pose significant challenges for fault diagnosis. Therefore, effectively monitoring and diagnosing mechanical faults has become a key issue in ensuring stable equipment operation, reducing maintenance costs, and preventing safety hazards [1,2,3,4].

To address this challenge, time–frequency analysis [5,6,7] has become a core tool in mechanical fault diagnosis. By decomposing vibration signals into their time and frequency components, time–frequency analysis (TFA) effectively captures how signal characteristics evolve over time and frequency, enabling the precise detection of early fault indicators.

TFA methods have undergone a gradual development from classical to innovative approaches. Early classical methods, such as Short-Time Fourier Transform (STFT) [8], Continuous Wavelet Transform (CWT) [9], and Wigner–Ville Distribution (WVD) [10], provided essential tools for signal analysis. However, these methods revealed limitations when dealing with complex nonlinear and non-stationary signals, as their time–frequency resolution was insufficient to accurately capture the dynamic characteristics of the signals. To enhance the precision and adaptability of TFA, researchers have proposed various innovative techniques based on traditional methods. The Smoothed Wigner–Ville Distribution (SWVD) [11] reduces cross-term interference caused by multi-component signals through smoothing processes, thereby improving time–frequency resolution. Empirical Mode Decomposition (EMD) [12] provides effective extraction of local oscillatory components in nonlinear and non-stationary signals through adaptive signal decomposition, while Hilbert–Huang Transform (HHT) [13] combines EMD with Hilbert Transform to further enhance the extraction of time–frequency features by capturing instantaneous frequency (IF). Adaptive STFT [14] dynamically adjusts the window function, and it can handle non-stationary signals. These innovative methods have enhanced the ability of TFA to handle complex signals, laying the foundation for future, more precise technologies.

Post-processing TFA methods aim to optimize existing time–frequency representations (TFRs), enhancing their readability, resolution, and accuracy. Initially, the reassignment method (RM) [15] was proposed to re-adjust the energy of the already generated TFR, reducing cross-term interference and improving the TFR. Subsequently, SST [16] and SRT [17] were introduced, which synchronized the optimization of the TFR, improving signal localization. Later, synchroextracting transform (SET) [18] and synchrosqueezing wavelet transform (SWT) [19] further advanced TFR optimization analysis of signals with multiple IF ridges. With the increasing demand for nonlinear signal analysis, second-order [20] and higher-order SSTs [21] were proposed, enhancing time–frequency feature extraction by utilizing higher-order spectral information. Additionally, Multi-synchrosqueezing transforms (MSSTs) [22,23] have been introduced to enhance the precision of TFRs. However, these methods rely on other TFA techniques to generate TFRs, while requiring these techniques to possess a certain level of signal processing capability, and face challenges such as high computational complexity and overlapping signal components.

Unlike post-processing methods based on TFR for ridge line re-extraction, Chirplet Transform (CT) [24] was proposed; it uses a chirp rate (CR) to rotate the orthogonal basis of STFT and achieves optimization of IF analysis. Afterward, several variants were proposed. In 2011, the self-tuning CT (STCT) [25] was introduced, which optimized window selection by incorporating a tuning mechanism, enhancing the flexibility of TFA. In the same year, Polynomial CT (PCT) [26] was proposed, which combined polynomial models to improve the accuracy of IF estimation for nonlinear signals. The Generalized Linear Chirplet Transform (GLCT) [27] extended the applicability of CT by incorporating a linear transformation framework, particularly suited for complex frequency-modulated signals. Synchronicity Compensation CT (SCCT) [28] addressed the frequency drift and time misalignment in multi-channel signals, improving the TFA accuracy of multi-source signals, although its computational load limits real-time processing capabilities. Velocity-Synchronized Linear CT (VSLCT) [29] optimized fault diagnosis for variable-speed machinery by synchronizing IF with rotational speed, showing exceptional performance in mechanical vibration analysis. Dual-Adapted CT [30] enhanced time–frequency resolution for radar signals through dual adaptations of time and frequency, although it performs less effectively for non-radar signals. Component-Matching CT (CMCT) [31] improved signal component extraction through frequency-dependent chirp rate adjustments, but its performance may be constrained when processing complex nonlinear signals. Velocity-Synchronized Chirplet Extraction Transform (VSCET) [32] enhanced the precision of signal extraction in variable-speed machinery, although its performance remains limited in complex signal environments. Although CT and its variants have demonstrated great potential in various fields, they still face challenges when processing signals with closely spaced or intersecting IF.

Currently, many researchers are exploring new TFA methods to more accurately describe and process IF signals with closely spaced, non-proportional, or crossing trajectories. The Adaptive Linear Chirplet Transform (ALCT) [33] was proposed, which dynamically adjusts the chirp rate to effectively handle signals with crossing frequencies and optimize instantaneous frequency estimation. However, this method may struggle to accurately resolve different frequency components in signals with small frequency separations. Y. He et al. introduced the entropy-matching Chirplet Transform with local maximum synchrosqueezing (EMCT) [34], which enhances the processing of crossing and nonlinear frequency signals through entropy matching, allowing for clear extraction of IF. However, EMCT comes with significant computational cost, especially in high-dimensional signal processing, and is sensitive to noise. Wu et al. proposed a General Chirplet Basis Transform (GCBT) [35], which combines different chirplet basis functions to enhance the analysis of multi-rotor vibration signals, particularly for signals with complex frequency modulation. A potential limitation of this method is that, when processing complex or noisy signals, artifacts may be generated, which could affect the accuracy of frequency component extraction. Afterward, the Local Optimal Scaling Chirplet Transform (LOSCT) [36] optimizes the scaling factor for processing non-stationary mechanical vibration signals with closely spaced frequencies, improving frequency resolution. However, its performance may degrade when handling highly nonlinear signals, and it requires high computational precision.

As mentioned above, existing TFA methods often fail to provide accurate and effective analysis results when analyzing signals with multiple IF ridges that are in close proximity and multiple components with different frequency bases. In the face of this situation, we consider a new approach—Local Entropy Optimization–Synchronous Demodulation Reassignment Transform (LEOADST). The main advantages of the LEOADST method include the following:

(1) The demodulation term is introduced, and by compensating for the CR, the time–frequency behavior of each component is adjusted, transforming each component of the signal into a stationary one;
(2) The local optimal theory based on Rényi entropy is introduced, with the TFR processed in blocks. The minimum entropy value of each sub-block is calculated to Identify the best demodulation parameters, thereby achieving precise matching of IF;
(3) Based on the maximum local energy criterion, the energy in the TFR is redistributed to obtain a more precise time–frequency distribution (TFD).

An overview of this paper’s organization is given here: In Section 2, we provide an overview of the Adaptive Synchronous Demodulation Transform (ASDT) [37] and explain the motivation behind proposing this new method. Section 3 introduces the Local Entropy Optimization–Adaptive Demodulation Reassignment Transform (LEOADRT) (LEOSDT), detailing its theoretical foundation and key features. The applicability and performance of LEOADRT are validated using the simulated signals in Section 4 and real mechanical signals in Section 5. Finally, Section 6 presents the conclusions, summarizing the proposed algorithm’s capabilities, applicability, and potential for practical industrial applications is provided.

## 2. Theoretical Basis and Research Motivation

### 2.1. Review ASDT

ASDT is developed from STFT. Li et al. [37] dynamically construct modulation components based on the frequency structure of the signal, making each component stationary simultaneously rather than adjusting the window size as in STFT. This approach eliminates the limitations of fixed window length, effectively improving time–frequency resolution and addressing the challenges in analyzing multi-component and nonlinear signals. This section will focus on the theoretical foundation of ASDT and provide a detailed analysis of how it overcomes the limitations of STFT, enhancing the ability to analyze complex signals.

For the components of the signal within each window, ASDT assumes them to be frequency-modulated signals and adaptively adjusts the demodulation term. This method effectively handles signals with nonlinear IF and significantly improves time–frequency resolution. Its specific form can be described by the following TFR equation:

(1) $$ASDT_{s}\left(f_{c},t_{c}\right)=\int_{-\infty }^{+\infty }s\left(t\right)D\left(l_{fun}\right)g\left(t-t_{c}\right)e^{-j2\pi f_{c}t}dt,$$

In the equation, *f_c_* and *t_c_* represent the central frequency and temporal position, while *g*(*t*) corresponds to a Gaussian function. The term *D(l*) is the demodulation term, defined as follows:

(2) $$D\left(l\right)=-0.5j\left[A\left(t\right)+B\left(t\right)f_{c}\right]{\left(t-t_{c}\right)}^{2},$$

In the equation, the demodulation terms hinge on the key parameters *A*(*t*) and *B*(*t*).

ASDT illustrates how to determine *A*(*t*) and *B*(*t*) in different signals. For the first type of signal, we first generate its phase function:

(3) $$\varphi_{i}\left(t\right)=\varphi_{i}^{\prime }\left(t_{c}\right)t+0.5\varphi_{i}^{\prime \prime }\left(t_{c}\right)\cdot {\left(t-t_{c}\right)}^{2},$$

where *φ_i_*″(*t_c_*) represents reflects the acceleration of the IF at time *t_c_*. If *φ_i_*″(*t_c_*) is constant, the parameter *B*(*t*) is determined to be zero. At this point, in the demodulation term of ASDT, only *A*(*t*) needs to be solved. By discretizing the angle into *N* discrete angles and utilizing the tangent function, a set of possible candidate values for *A*(*t*) can be obtained, as shown by the following equation:

(4) $$A_{k}=-\pi /2+\frac{k\pi }{N+1},$$

(5) $$\varphi_{i}^{\prime \prime }\left(t\right)=A\left(t\right)=A_{k}\left(t\right)=\tan\left(A_{k}\right),$$

In this case, by calculating the energy of the IF components at *t_c_* and selecting its minimum value, the optimal demodulation parameter *A*(*t*) can be determined. At that point, the parameter *A*(*t*) in the demodulation term is uniquely determined, and the energy *T* can be expressed as follows:

(6) $$T=\int_{0}^{f_{s}\cdot \pi }\left|ASDT_{s}\left(f_{c},t_{c}\right)\right|df=\sum_{i=1}^{n}0.5\pi \cdot A_{i}\left(t\right)\cdot \sqrt[{4}]{\left(\sigma^{4}{\left(\varphi_{i}^{\prime \prime }\left(t\right)-A_{k}\left(t\right)\right)}^{2}+1\right)},$$

where *A_i_*(*t*) serves as a measure of the amplitude for the *i*-th signal element at the specific instant *t*, and *A_k_*(*t*) is the demodulation parameter that needs to be solved for.

For signals with proportional IFs, the ASDT method can also be effectively applied. Specifically, the IFs of the pseudo-carrier signals in ASDT are proportional to each other, meaning that for each signal component, there exists a constant proportional factor. As a result, the demodulation parameter *A*(*t*) is determined to be 0, while *B*(*t*) is directly related to the proportionality of the IFs. To determine *B*(*t*), ASDT analyzes and optimizes the characteristics of the signal.

Similar to the approach for the first type of signal, solving for *B*(*t*) involves discretizing the angle range and using the tangent function to obtain a set of possible *B*(*t*) values. Then, using the same method, the optimal demodulation parameter *B*(*t*) is determined. Finally, by accurately determining the parameters A and B, we can obtain a clear TFR.

In addition to improving time–frequency resolution and handling nonlinear multi-component signals, ASDT also possesses the capability of signal reconstruction and modal decomposition. This feature of ASDT can decompose a complex signal into simpler components, with each component corresponding to a unique frequency component that has related IF and amplitude, as represented by the following equation:

(7) $$s\left(t\right)=\sum_{i=1}^{n}ASDT_{s}\left(\varphi_{i}^{\prime }\left(t\right),t\right)/\left(0.5\overset{⌢}{g}\left(0\right)\right),$$

(8) $$s_{i}\left(t\right)=ASDT_{s}\left(f_{c},t_{c}\right)/\left(0.5\overset{⌢}{g}\left(0\right)\right),$$

In the above expressions, *s_i_*(*t*) represents the *i*-th modal signal.

Next, we will describe the final implementation process of the ASDT algorithm, specifically demonstrating how ASDT uses these demodulation parameters and the associated window function to perform the decomposition of the signal in both time and frequency domains.

The core of this process lies in the combination of the demodulation term and the window function, which enables ASDT to achieve optimized time–frequency decomposition and obtain a more accurate time–frequency representation. The following equations will provide a detailed illustration of this implementation step:

(9) $$ASDT_{s}\left(f_{c},t_{c}\right)=\int_{-\infty }^{+\infty }s\left(t\right)e^{-0.5j\cdot a\left(t\right){\left(t-t_{c}\right)}^{2}}g\left(t-t_{c}\right)e^{-j\pi f_{c}\left(b\left(t\right){\left(t-t_{c}\right)}^{2}+2t\right)}dt,$$

It is further transformed into a discrete form:

(10) $$ASDT_{s}\left(f_{c}^{\prime },t_{c}^{\prime }\right)=\sum_{i=0}^{L-1}s\left(v\right)e^{-\pi ja\left(t_{c}^{\prime }\right){\left(v-t_{c}^{\prime }\right)}^{2}}g\left(v-t_{c}^{\prime }\right)e^{\frac{-\pi jf_{c}^{\prime }}{L}\left(b\left(t\right){\left(v-t_{c}^{\prime }\right)}^{2}+2v\right)},$$

In the equation, *f_c_*′ represents the discrete frequency center, which ranges from 0 to *L* − 1 as integers; *t_c_*′ represents the discrete time center.

### 2.2. Limitations of ASDT and Motivation

According to the analysis in Section 2.1, ASDT has certain advantages in handling complex signals, particularly nonlinear multi-component signals. However, when solving for the modulation parameters *A*(*t*) and *B*(*t*), ASDT assumes that the signal components satisfy specific proportional relationships, which can be categorized into two types: (1) Proportional frequency modulation signals: In this case, the frequency curvature (i.e., frequency modulation) is proportional to the frequency modulation of another signal, indicating a regular frequency variation, which allows the modulation parameters to be derived. (2) Proportional ratio signals in harmonic signals: For harmonic signals, ASDT assumes that the ratio of frequency modulation to the first derivative of the phase function is constant, which enables ASDT to accurately extract the modulation parameters.

Under these conditions, ASDT can effectively solve for *A*(*t*) and *B*(*t*). However, when the waveform consists of several independent fundamental frequencies in multi-component signals, the performance of ASDT significantly deteriorates, as it becomes difficult to effectively decompose the signal and accurately solve for the modulation parameters. This type of intricate signal can be represented by the following:

(11) $$N(u)=\sum_{n=1}^{L}\sum_{n=1}^{l_{j}}A_{ni}(u)e^{\left(-2\pi j\int v_{ni}f_{n}(u)du\right)}+\sigma (u),$$

The expression *N*(*u*) represents the total signal, which consists of multiple frequency components. The signal is composed of *L* base frequency components, with each base frequency component having *l_j_* subcomponents, and the amplitude of each component is described by *A_ni_*(*u*), and each frequency component is represented by *v_ni_*, and the frequency of the component varies with *u*, expressed as *f_n_*(*u*). The complex exponential term describes the variation in frequency and phase, representing the modulation process of the signal. Finally, *σ*(*u*) represents the noise term.

To further illustrate the limitations of ASDT and the motivation behind the development of our algorithm, we designed a set of simulation signals:

(12) $$x_{1}(u)=\sum_{k=1}^{6}a_{k}\cdot \sin\left(2\pi \int_{0}^{t}v_{k}(v)dv+\varphi_{k}\right)+\sigma (u),$$

In this signal expression, *a_k_* reflects the strength or amplitude of that frequency component; *v_k_*(*u*) denotes different IFs, describing how the frequency of that component changes with time *u*; and *φ_k_* is the initial phase of the different IF ridges, indicating the phase offset of the signal at *t* = 0. To better approximate real-world scenarios, we have added a noise term *σ*(*u*). Each signal component is expressed as follows:

(13) $$\left\{\begin{array}{c} v_{1}(u)=1/500\cdot {\left(u-28\right)}^{2}+1.2\\ \begin{array}{c} v_{2}(u)=1/1000\cdot {\left(u-28\right)}^{2}+0.5\\ \begin{array}{c} v_{3}(u)=3/1000\cdot {\left(u-28\right)}^{2}+2.2\\ v_{4}(u)=-1/600\cdot {\left(u-32\right)}^{2}+9 \end{array}\\ v_{5}(u)=0.9\cdot v_{4}(u)+0.6 \end{array}\\ v_{6}(u)=1.1v_{4}(u)-1.4 \end{array}\right.,$$

In this set of signals, the amplitude coefficients *a_i_* (*i* = 1,2, …,6) are set as 1, 2, 3.1, 5, 0.8, and 0.5, respectively. The phase shifts *φ_i_* (*i* = 1,2, …,6) are set as 0, π/12, π/5, 3π/2, π/4, and 2π/3, and we select 60 s of the signal for analysis.

To accurately assess the capabilities and limitations of the ASDT algorithm, six signal components were divided into three groups for analysis. The first experiment selected the first cluster of signals, consisting of frequency components *v*_1_, *v*_2_, and *v*_3_, denoted as *S*_1_, which exhibit a clear proportional relationship. The second experiment selected the second cluster of signals, consisting of frequency components *v*_4_, *v*_5_, and *v*_6_, denoted as *S*_2_. The frequency components within this cluster have relatively small intervals, representing near-intermediate frequency signals. Lastly, the third experiment combined all the frequency components from both the first and second clusters, i.e., the composite signal *S*_3_ containing *v*_1_, *v*_2_, *v*_3_, *v*_4_, *v*_5_, and *v*_6_. This experiment aims to evaluate the performance of ASDT in handling non-proportional frequency components, especially when both clusters of signals coexist, verifying whether ASDT can effectively separate and analyze these frequency components.

**Figure 1:**
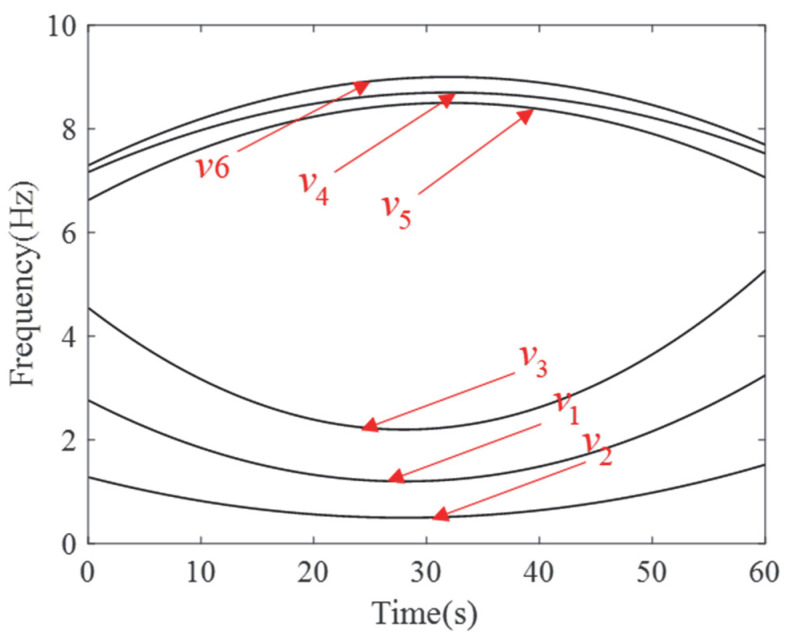
Ideal IFs of six frequency components.

**Figure 2:**
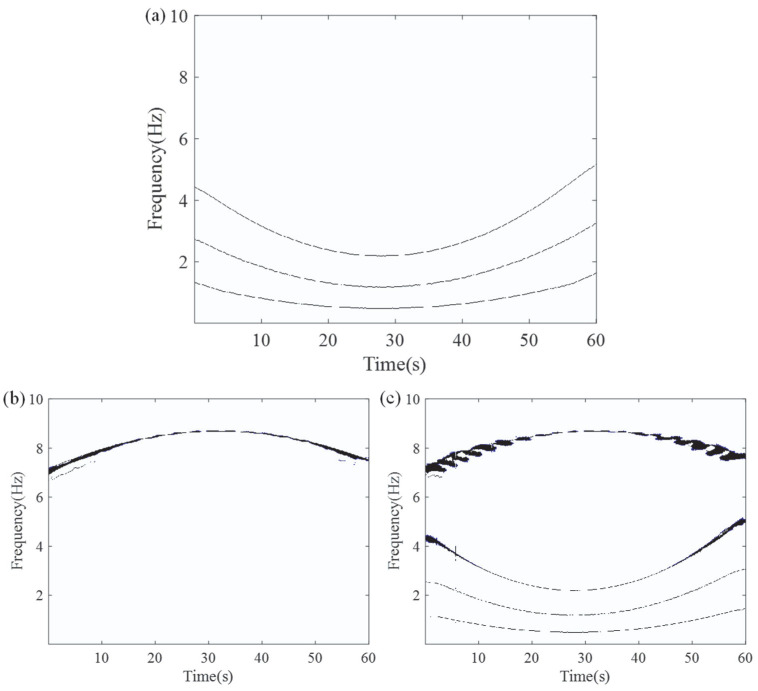
TFR of ASDT analysis for the three experiments: (**a**) Experiment 1, (**b**) Experiment 2, and (**c**) Experiment 3.

The ideal IF plot of the six frequency components is shown in Figure 1. The results of the ASDT algorithm from three experiments are presented in Figure 2a–c. The analysis results indicate that ASDT effectively analyzes nonlinear multi-component signals, providing clear TFRs and a high degree of energy concentration. However, ASDT is unable to effectively analyze the signal with closely spaced frequencies and non-proportional IF components.

In summary, the drawbacks of ASDT and the motivation for the research can be summarized as follows:

(1) When the IF trajectories of the analyzed signal are very close to each other, ASDT fails to accurately analyze their time–frequency characteristics. This is due to the frequency overlap that occurs when the components are close together, which impairs ASDT’s capacity to distinctly isolate and differentiate separate frequency elements, thereby reducing time–frequency resolution;
(2) ASDT struggles to represent nonlinear signals with multiple base frequencies. Specifically, when the IFs of the target signal are proportional, ASDT provides the optimal TFR. However, when the frequency function of the signal is non-proportional, the demodulation process in ASDT cannot accurately solve for the IFs, thus limiting its ability to effectively analyze non-proportional IF signals.

Therefore, there is a pressing need to develop a new TFA algorithm capable of being broadly applied to time-varying signal analysis, especially those with non-proportional intermediate frequencies or closely spaced IF characteristics, offering improved resolution and accuracy.

## 3. Local Entropy Optimization–Adaptive Demodulation Reassignment Transform

The Local Entropy Optimization–Synchronous Demodulation Transform (LEOADST) is an extension of the ASDT framework. This section provides an in-depth exploration of the core mathematical theory underlying LEOADST, with a focus on the local optimization method based on Rényi entropy and its energy redistribution strategy.

### 3.1. Local Optimization Using Rényi Entropy

The core idea behind the local optimization using Rényi entropy is to adjust the scaling factor of the chirplet basis to quantify how the signal’s energy is spread across the TFR, thereby optimizing the signal’s time–frequency characteristics. Specifically, Rényi entropy selects the optimal scaling factor to enhance the clarity of the TFR, making the IF components within the signal more prominent and easier to identify.

Rényi entropy offers a way to quantify how the signal’s energy is concentrated, with its minimum value indicating that the TFR accurately reflects the IF components of the signal. To further quantify this process, the definition of the α-order Rényi entropy is introduced:

(14) $$R^{\alpha }=\frac{1}{1-\alpha }\log_{2}\frac{\iint T^{\alpha }\left(\varphi ,\gamma \right)d\varphi d\gamma }{\iint T\left(\varphi ,\gamma \right)d\varphi d\gamma },$$

In the equation, *T*(*φ*, *γ*) represents the TFR; the parameter *α* is typically set to 3.

**Figure 3:**
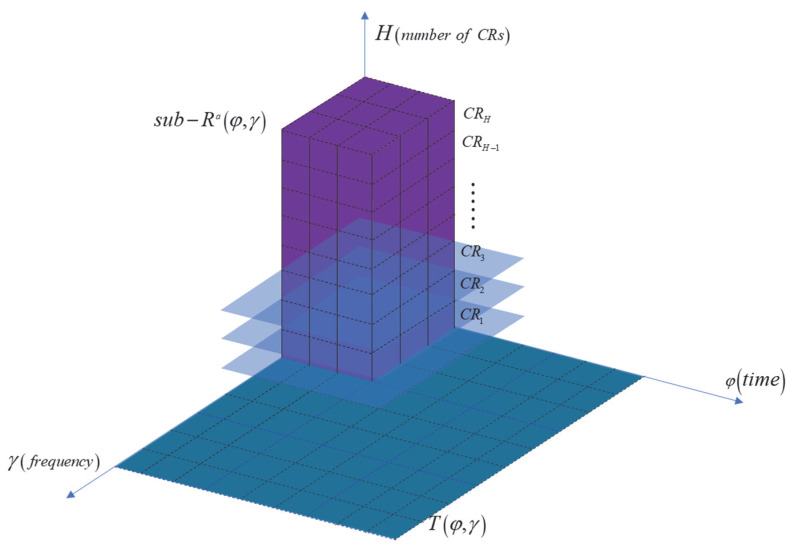
Time–frequency block segmentation and the Rényi entropy calculation.

Next, we divide the time–frequency domain into multiple regions to more precisely evaluate the signal’s behavior in the time–frequency domain. Specifically, through shifting the *W*(*φ*, *γ*) across both the temporal and frequency directions, we can divide the time–frequency representation *T*(*φ*, *γ*) into smaller blocks. As shown in Figure 3, this process continues until the entire time–frequency plane is covered. Then, the Rényi entropy of each time–frequency block is computed to quantify the energy concentration of the signal within each block. The division of the blocks and the calculation of entropy are expressed as follows:

(15) $$R\left(c,\varphi ,\gamma \right)=\frac{1}{1-\alpha }\log_{2}\left(\int_{-\infty }^{+\infty }\int_{-\infty }^{+\infty }{\left(\frac{\left|T\left(c,\xi ,\eta \right)W\left(\xi -\varphi ,\eta -\gamma \right)\right|}{\int_{-\infty }^{+\infty }\int_{-\infty }^{+\infty }\left|T\left(c,\xi ,\eta \right)\right|W\left(\xi -\varphi ,\eta -\gamma \right)d\xi d\eta }\right)}^{\alpha }d\xi d\eta \right),$$

(16) $$W\left(\varphi ,\gamma \right)=\left\{\begin{array}{c} \;1,-0.5\Delta \varphi <\varphi <0.5\Delta \varphi ,-0.5\Delta \gamma <\gamma <0.5\Delta \gamma \\ 0,\;\;\;\;\;\;otherwise \end{array}\right.,$$

where *R*(*c*, *φ*, *γ*) calculates the entropy values for different regions, *T*(*c*, *ξ*, *η*) is the TFD in each block, *W*(*ξ* − *φ*, *η* − *γ*) represents the function that determines the dimensions of the time–frequency region, and Δ*φ* and Δ*γ* represent the block size in both the time and frequency dimensions, respectively.

By introducing a series of discrete CRs, the signal is transformed into a 3D representation, *H* × *L* × *L*/2, denoted as *T*(*c*, *φ*, *γ*), where *H* represents the number of different CRs, corresponding to H distinct TFRs, and *L* is the discrete length of the signal, determining the time–frequency resolution. The entire TFR is divided into *W_φ_* × *W_γ_* time–frequency blocks, and the entropy is calculated using (14), thus reducing the dimensionality of the three-dimensional space to *H* × *W*_φ_ × *W*_γ_. Each time–frequency block contains *H* entropy values, and we calculate the required minimum entropy using the following equation:

(17) $$c_{0}\left(\varphi ,\gamma \right)=\underset{c}{\arg\min}\left\{R\left(c,\varphi ,\gamma \right)\right\},$$

**Figure 4:**
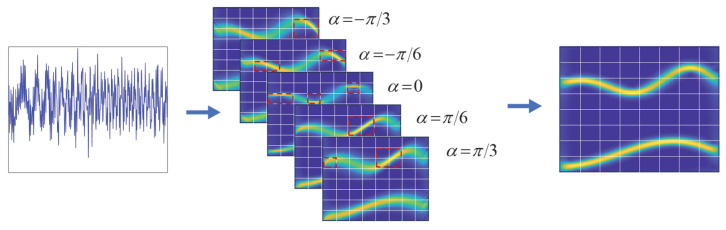
Example of the principle of local Rényi entropy optimization (*H* = 5, *φ* = 8, *γ* = 8).

We select the demodulation parameters corresponding to the minimum entropy. Next, using the *W*_φ_ × *W*_γ_ method, we filter the time–frequency blocks that exhibit the highest energy concentration and recombine these blocks to obtain an enhanced two-dimensional TFR with a size of *L* × *L*/2. Finally, based on the optimization strategy of the two-dimensional Rényi entropy, we obtain an enhanced TFR, as shown in Figure 4. In conclusion, by introducing Rényi entropy optimization to adjust the demodulation parameters in the ASDT demodulation process, we can select the most suitable demodulation parameters for each time–frequency block; consequently, this greatly enhances the accuracy of signal demodulation. The proposed LEOADT method is described as follows:

(18) $$LEOADT_{c_{0}}\left(\varphi ,\gamma \right)=T\left(c_{0},\varphi ,\gamma \right),$$

### 3.2. Ridge Enhancement and Time–Frequency Energy Concentration Mechanism

In this section, we will explore methods to tackle the energy leakage issue when studying highly dynamic AM-FM signals by introducing a one-dimensional IF estimation principle based on LEOADT, thus enhancing the clarity and energy concentration of LEOADT. The frequency reassignment operator redistributes the energy, ensuring a more precise TFR for the signal, reducing energy spread, and enhancing the signal’s demodulability. The core goal of frequency reassignment is to enhance the structural clarity of the signal, making the time–frequency distribution more concentrated. Next, we will present the specific formula for the frequency reassignment operator:

(19) $$LEOADT_{c_{0}}\left(\varphi ,\gamma \right)=T\left(c_{0},\varphi ,\gamma \right),$$

The Gaussian window function, being symmetric, exhibits a maximum magnitude in its Fourier transform at zero frequency. Therefore, by utilizing the symmetry inherent in the Gaussian window function to constrain the range of frequency spread, the frequency redistribution operator (RO) could be reformulated as follows:

(20) $$f_{r}\left(\varphi ,\gamma \right)=\left\{\begin{array}{c} f_{inst}(\varphi ),if\;f_{\varphi }\in \left[f_{inst}(\varphi )-\Delta ,f_{inst}(\varphi )+\Delta \right]\\ 0,\;\;\;\;\;\;\;\;\mathrm{otherwise}\; \end{array}\right.,$$

This expression precisely controls the redistribution of energy, making the signal’s TFR clearer and effectively reducing energy leakage, thereby enhancing the demodulation accuracy and analyzability of the signal. Based on this, the RO is further expressed as LEOADT–O:

(21) $$LEOSDT_{c_{0}}-O(\varphi ,\gamma )=\delta (\varphi -f_{r}(\varphi ,\gamma ))=\left\{\begin{array}{c} \;1,\left|\varphi -f_{r}(\varphi ,\gamma )\right|<\eta \\ 0,\;\;\;\;\;\;\;otherwise \end{array}\right.,$$

In the equation, *η*(⋅) denotes the Dirac delta function. In order to enable the LEOADT–O to approximate the frequency center, we introduce a small parameter *η*, which relaxes the constraints appropriately to achieve this goal.

This parameter plays a critical role in the frequency reassignment process, as it defines the allowable deviation from the frequency center. Specifically, when the frequency difference is less than *η*, the corresponding frequency component is considered sufficiently close to the center and is thus eligible for energy reassignment. In this way, *η* functions as a soft threshold, effectively filtering out irrelevant components and enhancing energy concentration in the TFR.

The design of *η* achieves a balance between theoretical precision and practical applicability, improving the flexibility and robustness of the reassignment process across different signal types. The optimal selection of *η* depends on the characteristics of the signal. A smaller *η* is preferable for signals with tightly clustered frequency components and high resolution requirements, while a larger *η* is more suitable for signals with broader frequency variations, as it allows more frequency components to contribute to the reassignment. Based on extensive empirical evaluation across multiple signal types, a value of *η* = 0.4 has been found to be an effective compromise, offering a favorable trade-off between accuracy and stability.

Following the previous analysis, we introduce an effective TFA method—LEOADRT. This method effectively improves time–frequency resolution and resolves the energy leakage issue by introducing local entropy optimization and the frequency reassignment operator. By precisely controlling frequency matching and energy distribution, LEOADRT provides a flexible and robust tool, particularly suitable for analyzing complex signals and multiple frequency components.

(22) $$LEOADRT\left(\varphi ,\gamma \right)=LEOADRT_{c_{0}}\left(\varphi ,\gamma \right)\cdot LEOADRT_{c_{0}}-O(\varphi ,\gamma ),$$

Algorithm 1 describes the LEOADRT algorithm.

**Algorithm 1.** The fundamental logic behind LEOSDRT


**LEOADRT Approach for TFA**

**Step 1: Given input parameters**

(1)Signal *x*(*t*), total count for the discrete angles *N,* sampling frequency *fs*, smoothing length *S*, window size *N_w_*, thresholds *η* and *σ*, and the number of vertical and horizontal blocks *W* and *D* and factor *O*.(2)*win_signal*←zeros(*L_x_*_(*t*)_, *N_w_*).(3)Split the range between −π/2 to π/2 across *N* discrete angle values:

$a_{k}=-\pi /2+\frac{k\pi }{N+1},k\in \left\{1,2,\ldots ,N\right\}$


**Step 2: LEOADT**

(4)**for** *j* = 1: *N* *sub*-*tfr*(:, :, *j*) ← T(*f_c_*′, *t_c_*′);

**end for**

(5)Use (15)–(17) to obtain the best angle for retrieval
**for** *w* = 1: *N*
 LEOADT (:, :) ← min(Rényi (*sub*-*tfr* (:, :,*w*)))
 TFR(:,*w*) ← sub-tfr(:, *w*, index);

**end for**


**Step 3: LEOADRT**

(6)Calculate: LEOADRT←zeros(*N_H_*, *N_H_*), LEOADT–O←zeros(*N_H_*, *N_H_*)(7)**for** *u* = 1: *N_H_*
 **for** *v* = 1: *N_H_*
  **if** *abs*(*u*- *fr*(*u*,*v*)) < *η*
   LEOADT–O (*u*,*v*)=1
  **end if**
 **end for**

**end for**

(8)Output: LEOADRT (*φ, γ*)


## 4. Simulation Signal Experiment

To validate the superiority of the proposed method, two simulation-based experiments are carried out in this part. The focus will be on evaluating its performance in handling non-stationary signals, signals with non-proportional components, and signals with closely spaced frequency components. Additionally, a comparison with other TFA methods will be made to further demonstrate the excellent performance of the LEOADRT method in analyzing such complex signals.

### 4.1. Non-Stationary Signals with Multiple Fundamental Components

First, we construct the signal according to the following formula:

(23) $$\begin{array}{l} x_{1}(t)=8\cdot sin(2\pi \cdot (1/500\cdot (t-15)2+5)\cdot t+\pi /6)\\ \;+5\cdot sin\left(32+(10\pi \cdot sin(2\pi \cdot t/T))\cdot t+\pi /5\right)\\ \;+7\cdot sin(15+(10\pi \cdot cos(2\pi \cdot t/T))\cdot t+10\pi /3)\\ \;+9\cdot sin(2\pi \cdot (1/500\cdot (t-15)2+6)\cdot t+\pi /4)\\ \;+\sigma \left(t\right) \end{array},$$

**Figure 5:**
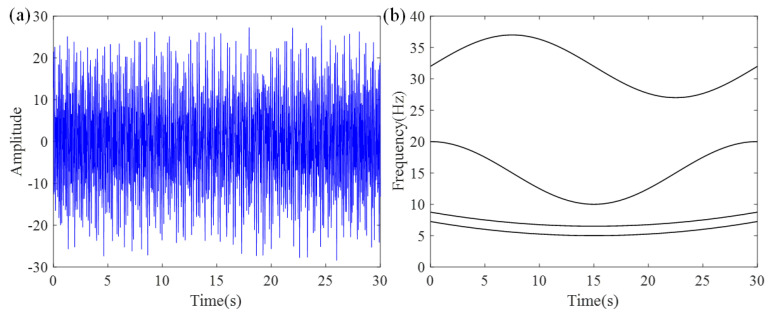
(**a**) Signal *x*_1_(*t*) waveform and (**b**) ideal TFR.

In this equation, *T* represents the analysis duration of 30 s, and *σ* denotes the noise term. To simulate a realistic environment, white noise is introduced with an SNR value of 10. The signal *x*_1_(*t*) is sampled at a frequency of 100 Hz, with its waveform and the ideal TFR displayed in Figure 5a and Figure 5b, respectively.

In the proposed LEOADRT method, the size of the time–frequency block *W*_φ_ × *W*_γ_ plays a critical role in determining the analysis performance. This parameter not only influences the resolution of the resulting TFR but also affects the computational efficiency to a certain extent. To thoroughly investigate its impact and provide a theoretical basis for optimal parameter selection, a series of systematic experiments are conducted.

To quantitatively assess the quality of TFRs generated under different time–frequency block configurations, a novel objective evaluation metric is introduced in this study, termed the Concentration Measure (*CM*). This metric evaluates the degree to which energy is concentrated in the high-intensity regions of the TFR. From a global perspective, it characterizes the sharpness and focus of the TFR, offering strong interpretability and method independence.

The definition of *CM* is given as follows:

(24) $$CM=\frac{\sum_{\left(\varphi ,\gamma \right)\in S}{\left|LEOSDT_{c_{0}}\left(\varphi ,\gamma \right)\right|}^{2}}{\sum_{\left(\varphi ,\gamma \right)}{\left|LEOSDT_{c_{0}}\left(\varphi ,\gamma \right)\right|}^{2}},$$

Here, *S* denotes the set of time–frequency points corresponding to the top *k*% largest magnitudes in the TFR, where *k* is set to 10 in this study.

To systematically investigate the impact of time–frequency block sizes on the *CM* metric, fourteen different block sizes ranging from 1 × 1 to 32 × 32 were selected. The first eight sizes (from 1 × 1 to 8 × 8) were incremented uniformly with a step size of 1 to obtain a fine-grained observation within this range. The remaining six sizes (from 12 × 12 to 32 × 32) were incremented with a step size of 4. This stepped sampling strategy was adopted based on preliminary experiments, which indicated that the *CM* metric is more sensitive and discriminative within the smaller block size range (approximately 1 to 10). Larger block sizes, although sometimes yielding relatively high *CM* values, tend to produce time–frequency representations with reduced resolution, thus limiting their interpretability and practical utility. Therefore, a sparser sampling with larger increments was used for the larger block sizes to balance computational efficiency and accuracy.

**Figure 6:**
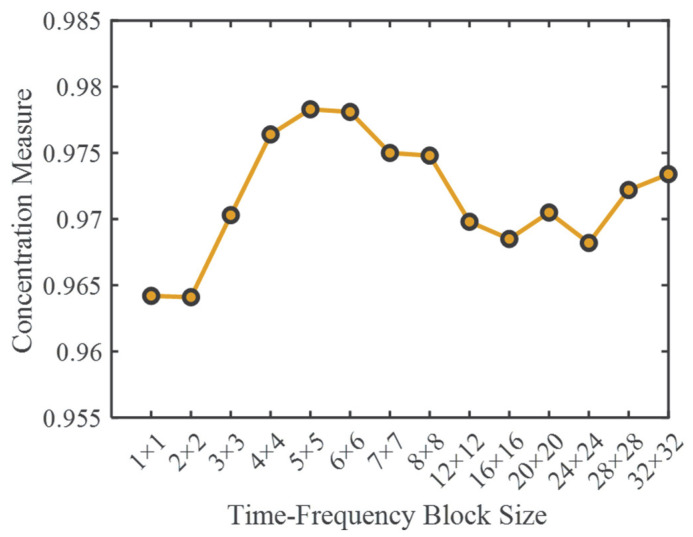
Impact of time–frequency block dimensions on *CM*.

The experimental results are shown in Figure 6, illustrating a clear variation in *CM* values with changing block sizes. Notably, the block size of 5 × 5 yielded the highest *CM* value, indicating the most concentrated energy and strongest focusing in the time–frequency representation, thus demonstrating superior time–frequency characterization capability.

**Figure 7:**
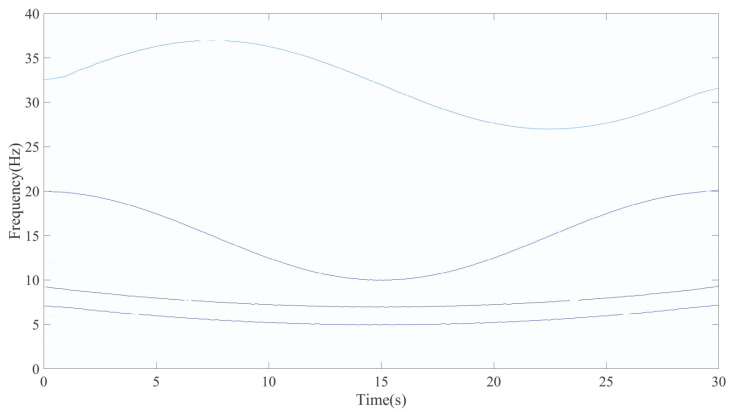
The TFR generated by analyzing the signal *x*_1_(*t*) using LEOADRT.

Based on this analysis, a block size of 5 × 5 was selected as the default configuration for subsequent experiments. The corresponding time–frequency representation generated with this block size is presented in Figure 7. It is important to note that signal properties such as signal length and instantaneous frequency intervals significantly influence the optimal choice of time–frequency block size. Hence, the selection of time–frequency block sizes needs to be discussed individually for different types of signals.

**Figure 8:**
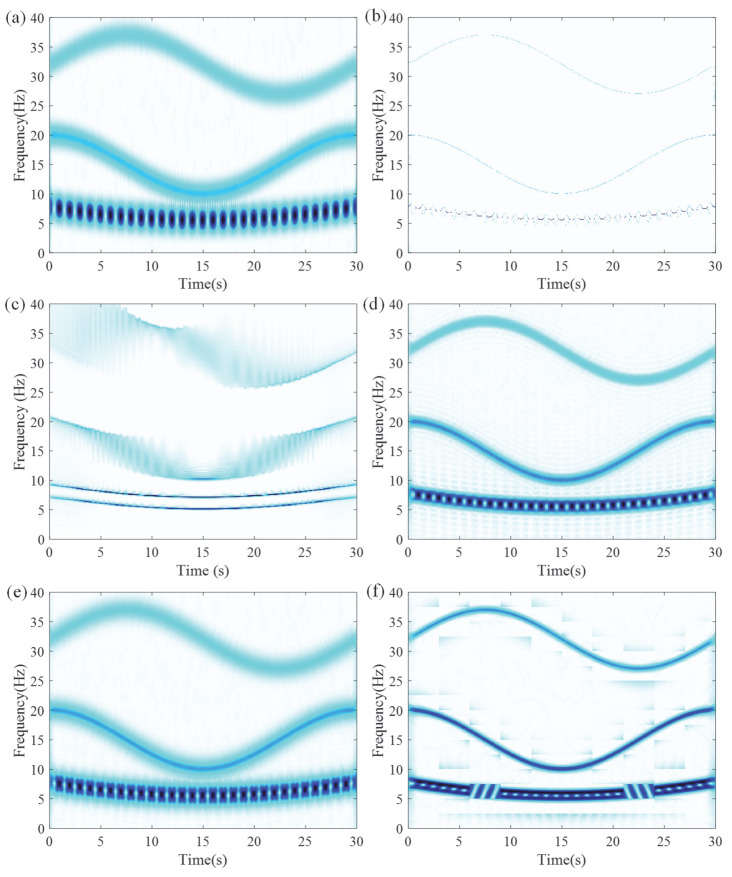
TFRs using different methods: (**a**) STFT, (**b**) SET, (**c**) VSLCT, (**d**) SBCT, (**e**) GLCT, and (**f**) EMCT.

As shown in Figure 7, the TFR generated by LEOADRT clearly displays the IF components with concentrated energy and high time–frequency resolution. To accurately evaluate the performance of LEOADRT, we analyze the signal *x*_1_(*t*) using six different methods: STFT, SET, VSLCT, SBCT, GLCT, and EMCT. Figure 8a–f display the corresponding TFRs.

As shown in Figure 8a–f, STFT, SBCT, VSLCT, and GLCT show limited effectiveness when dealing with signals composed of multiple components and fundamental frequencies. The resulting TFRs suffer from noticeable energy dispersion and relatively poor time–frequency resolution. Furthermore, SET, STFT, SBCT, and EMCT demonstrate difficulty in accurately characterizing signal components with closely spaced frequencies, leading to challenges in resolving their respective time–frequency features.

To additionally assess the performance of the LEOADRT approach in handling complex time-varying signals and to provide an objective evaluation of TFR quality produced by various TFA techniques, two widely adopted image quality assessment metrics are employed: Peak Signal-to-Noise Ratio (PSNR) and Structural Similarity Index (SSIM). Specifically, PSNR quantifies the pixel-wise error between the generated TFR and the ideal TFR, with higher values indicating better reconstruction fidelity. In contrast, SSIM evaluates the perceptual similarity between TFRs by considering luminance, contrast, and structural information, thereby providing a more comprehensive and human-vision-aligned assessment of structural consistency.

**Figure 9:**
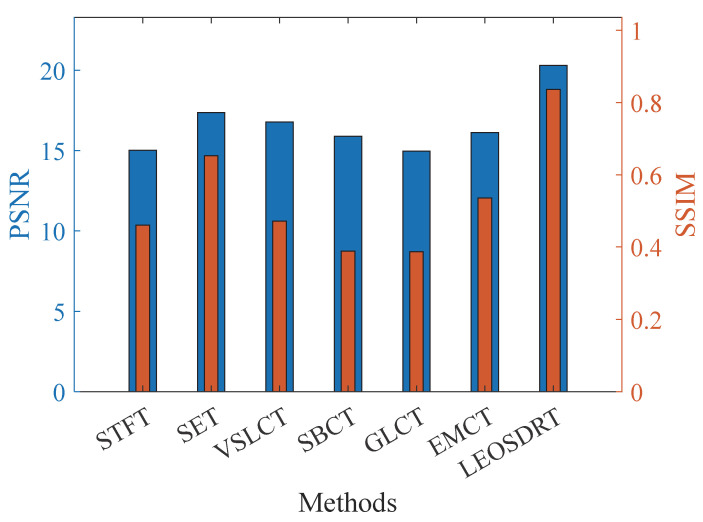
Comparison of PSNR and SSIM values for different methods.

The PSNR results for signal *x*_1_(*t*), derived using various methods, are presented in Figure 9 by the left Y-axis and the blue bars. From the figure, it is evident that LEOADRT performs the best among all methods, achieving the highest PSNR value. This indicates that LEOADRT has a significant advantage in preserving time–frequency structural details and minimizing distortion. In contrast, the PSNR values for GLCT and STFT are relatively low, reflecting significant information loss or noise interference in the signal processing task. Additionally, the SSIM values, shown by the right Y-axis and the orange bars in Figure 9, further support this observation. LEOADRT generates a TFR with the highest structural similarity to the ideal TFR, indicating its ability to more accurately analyze complex signals with non-proportionality and closely spaced IF trajectories. It also accurately retains the signal’s time–frequency characteristics and exhibits higher structural integrity.

### 4.2. Analysis of Cross-Interacting Non-Stationary Signals

A cross-component signal comprising multiple components is designed in the following manner:

(25) $$\begin{array}{l} x_{2}(t)=10\cdot sin(2\pi \cdot (10\cdot \cos(\pi \cdot t/T)+20)\cdot t+\pi /6)\\ \;+7\cdot sin\left(2\pi \cdot (6\cdot ones\left(size\left(t\right)\right)+32)+\pi /5\right)\\ \;+\sigma \left(t\right) \end{array}$$

**Figure 10:**
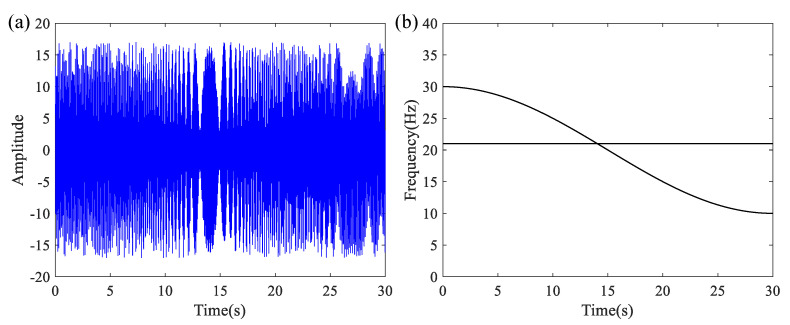
(**a**) Time–domain signal *x*_2_(*t*) and (**b**) its corresponding ideal TFR.

**Figure 11:**
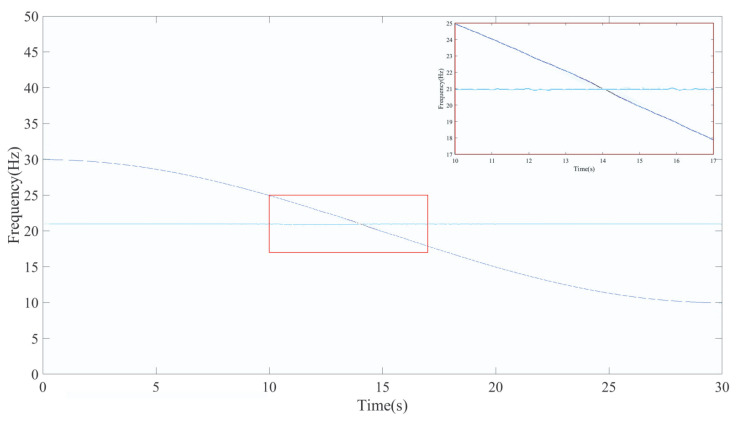
TFR using LEOADRT analysis.

A window length of 200 is adopted, while the remaining parameters follow the configuration used for signal *x*_1_(*t*) in Section 4.1. Figure 10a,b illustrate the time-domain representation of *x*_2_(*t*) and its corresponding ideal IF curve. The TFR of signal *x*_2_(*t*) obtained using the proposed LEOADRT method is displayed in Figure 11.

**Figure 12:**
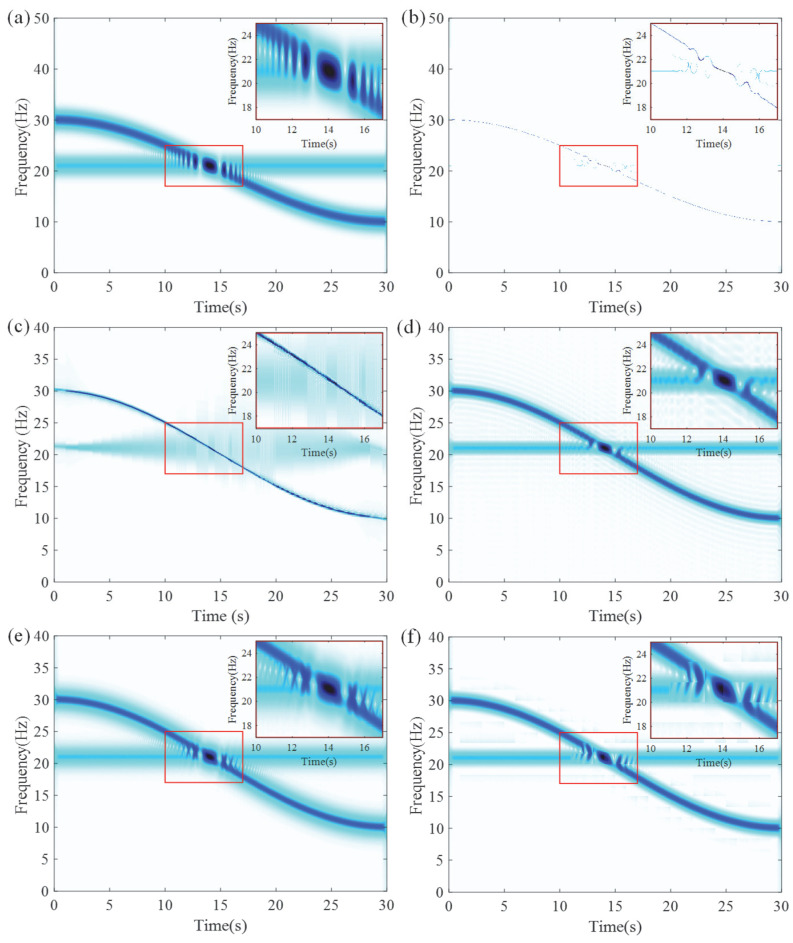
Analysis results for signal *x*_2_(*t*) using different techniques: (**a**) STFT, (**b**) SET, (**c**) VSLCT, (**d**) SBCT, (**e**) GLCT, and (**f**) EMCT.

From Figure 11, it can be observed that the time–frequency resolution of the IF curves is clear, and the energy concentration in the crossing region is relatively high. The zoomed-in view also shows clear and continuous IF curves. For comparison, we further analyze signal *x*_2_(*t*) using STFT, SET, VSLCT, SBCT, GLCT, and EMCT. All parameters and settings are kept consistent with the previous analysis. Figure 12 presents the results of these methods, from which it is evident that the STFT, SBCT, GLCT, and EMCT methods fail to accurately represent the IF curves in the crossing region, with noticeable energy divergence in this area. The SET method exhibits poor time–frequency resolution in the crossing region and is unable to clearly resolve the signal’s individual frequency components. The VSLCT method fails to accurately represent the horizontal components and shows significant energy divergence in the crossing region.

**Figure 13:**
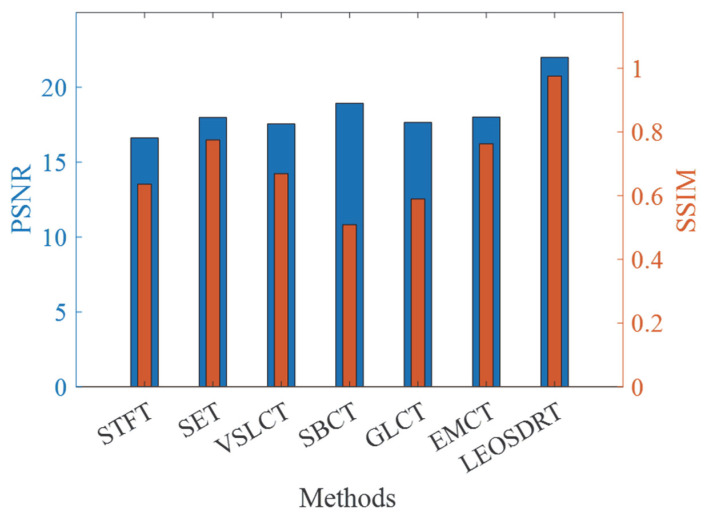
Comparison of PSNR and SSIM across different methods.

In order to thoroughly confirm the superiority that the LEOADRT approach demonstrates in analyzing cross-component signals compared to other mainstream methods, PSNR and SSIM were used to quantitatively evaluate the results. Figure 13 displays the PSNR and SSIM comparison results, respectively. As indicated by the left Y-axis and the blue bars shown, the LEOART method exhibits the highest PSNR, significantly outperforming other methods, indicating its superior fidelity in time–frequency reconstruction. The SSIM comparison results, shown by the right Y-axis and the orange bars, further corroborate this, with the proposed method’s generated TFR being closest to the ideal value of 1, indicating the highest structural similarity to the original signal.

In summary, through the constructed signal sets and comparisons with other mainstream TFA methods, it is demonstrated that the LEOADRT method possesses a notable edge in the analysis of non-stationary signals, especially in cases of non-proportional and closely spaced components. The generated TFR exhibits higher energy concentration and better time–frequency resolution, making it highly suitable for the TFA of complex signals.

## 5. Experimental Assessment

Through subsequent experiments, the LEOADRT approach is shown to be both efficient and advantageous in analyzing complex non-proportional or cross-coupled signals using three representative mechanical signal datasets. The first dataset consists of gearbox and gear vibration signals with non-proportional rotational speeds, the second dataset includes real-world cross-coupled vibration signals from a hydroelectric turbine unit, and the third dataset features experimental signals from an inner ring bearing fault. These signal sets were carefully selected to cover various engineering application scenarios, focusing on thoroughly demonstrating how the LEOADRT method performs in practical industrial signal processing scenarios.

### 5.1. Non-Synchronous Characteristics of Gearbox and Fault Bearing Signals on a Test Rig

**Figure 14:**
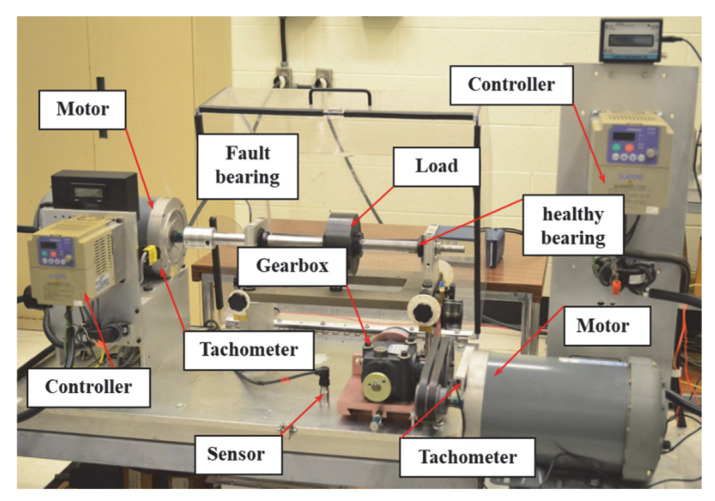
Diagram illustrating the test system and supporting equipment.

The first set of experiments involves the analysis of signals with mismatched rotational speeds, consisting of two distinct signal sources. The first signal is derived from a faulty bearing driven by the left-side motor, while the second signal originates from a gearbox driven by the right-side motor. The bearing’s fault characteristic frequency (FCF) is associated with a frequency component located at 3.854 × *f_r_*, where *f_r_* denotes the rotational frequency (RF) [38]. The experimental setup and related equipment are shown in Figure 14.

**Figure 15:**
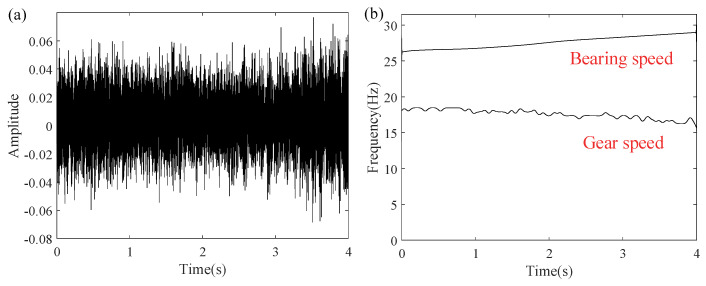
(**a**) Time–domain waveform of the measured vibrations and (**b**) associated RF signal variation.

The system operated under varying conditions, with bearing speed accelerating to 29 Hz from an initial 23 Hz, while the gearbox exhibited a 4.4 Hz decline (from 20.6 Hz to 16.2 Hz). Given the extended duration of the experimental signals, a 4 s segment was extracted and resampled at 1000 Hz for further processing. For the selected time segment, Figure 15a,b present the vibration waveform and corresponding RFs.

**Figure 16:**
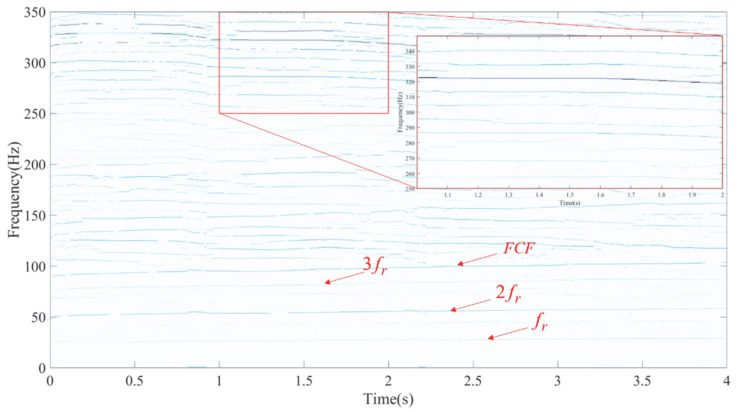
TFA results using the LEOADRT method.

**Figure 17:**
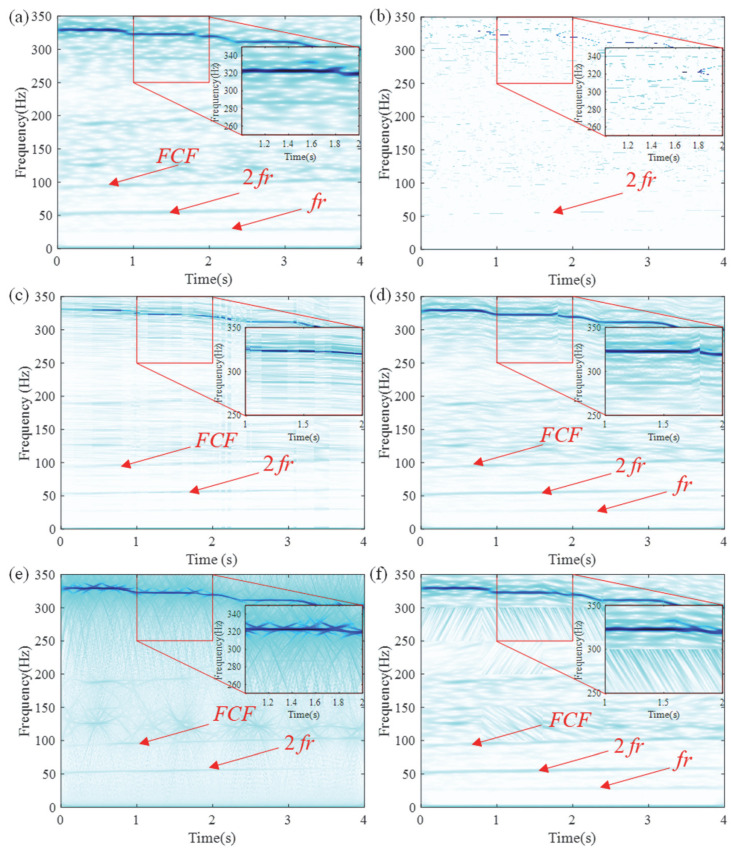
TFA comparison of six methods: (**a**) STFT, (**b**) SET, (**c**) VSLCT, (**d**) SBCT, (**e**) GLCT, and (**f**) EMCT.

Using the LEOADRT approach, the signal was analyzed, with the resulting TFR displayed in Figure 16. The FCF and various harmonics of the fault bearing signal, such as *f_r_*, 2 *f_r_*, 3 *f_r_*, etc., are clearly observed in the TFR. In the high-frequency region, a localized zoom-in reveals distinct IF ridges, indicating high time–frequency resolution. To provide additional evidence of LEOADRT’s effectiveness, a comparative analysis was carried out, analyzing the same signal using six different methods: STFT, SET, VSLCT, SBCT, GLCT, and EMCT. The TFRs for these techniques are displayed in Figure 17a–f.

**Figure 18:**
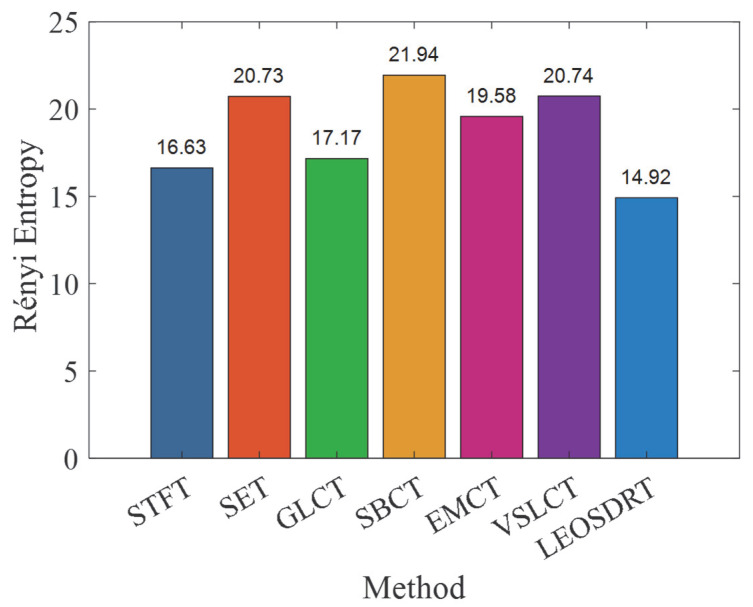
Comparison of Rényi entropy values for different methods.

From the TFRs, it can be seen that while all methods can effectively identify low-frequency bearing fault signal components and the FCF, the gearbox signal in the high-frequency region shows varying degrees of energy dispersion, resulting in lower time–frequency resolution. Notably, the IF ridges extracted by SET and GLCT methods are severely affected by interference. In contrast, the IF estimation for the gearbox vibration signal is also somewhat blurred in the EMCT and VSLCT methods. To evaluate the effectiveness of these methods, the Rényi entropy was calculated for all six approaches, as shown in Figure 18. Among the methods tested, the LEOADRT approach exhibits the lowest entropy, with a value of 15.9232, while the GLCT method has the highest value of 21.9433. This demonstrates that the LEOADRT method produces a TFR that concentrates more energy, which is consistent with the visual observations from the TFR.

To evaluate the computational complexity and runtime efficiency of the proposed LEOADRT algorithm, we conducted a comparative experiment using signals from a bearing gear system with asynchronous rotational speeds. Six existing methods were compared against our algorithm in terms of computation time. The experiments were performed on the following hardware: Intel Core i9-14900HX processor (32 cores, 2.2 GHz) (Intel, Santa Clara, CA, USA), equipped with Intel UHD Graphics (2GB) (Intel, Santa Clara, CA, USA) and NVIDIA GeForce RTX 4060 Laptop GPU (8 GB), 32 GB DDR5 5600 MHz RAM (NVIDIA, Santa Clara, CA, USA), a 2 TB SSD, and MATLAB 2024b as the software environment.

As shown in Table 1, LEOADRT demonstrates higher computational efficiency than EMCT, VSLCT, and GLCT. Although its runtime is slightly longer than traditional time–frequency analysis methods such as STFT and SET, LEOADRT exhibits significant advantages in the quality of the TFRs. Specifically, the TFDs produced by LEOADRT offer higher resolution and better energy concentration, revealing more detailed signal features and clearer frequency characteristics.

In summary, the computational efficiency of LEOADRT is acceptable, and it achieves notable improvements in extracting time–frequency features from signals, demonstrating its practical value in the analysis of complex signals.

### 5.2. Characterizing Crossed IFs in Turbomachinery Vibration Signals

**Figure 19:**
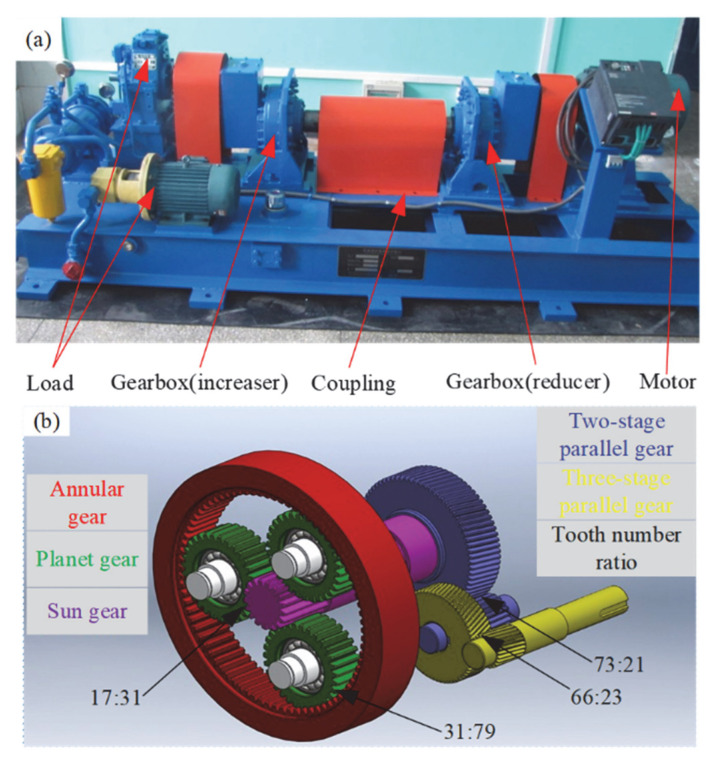
Diagram of the turbine planetary gear system structure and gear ratio: (**a**) experimental setup diagram; (**b**) gear system structure and gear ratio.

**Figure 20:**
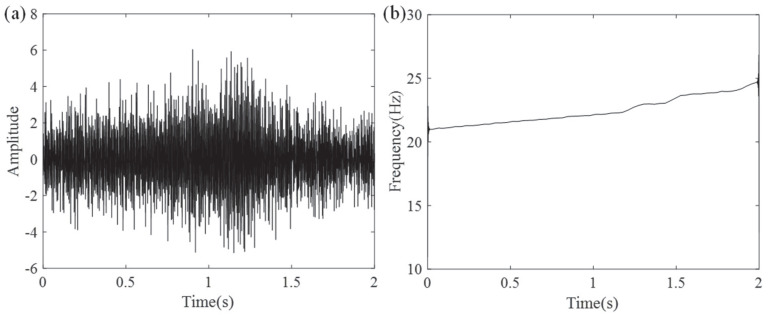
(**a**) Waveform of the measured vibrations and (**b**) associated RF signal variation.

In the second set of experiments, we conducted an in-depth analysis of the actual mechanical vibrations produced by the planetary gear system of the turbine during operation. The IF ridges of the signal exhibited significant crossing phenomena. Figure 19a,b present the configuration of the experimental system and the associated components in detail as the gear system’s components and their gear ratios. To reduce data volume and improve analytical efficiency, we downsampled the original signal at 720 Hz, Figure 20 presents the output results, where subfigure (a) shows the waveform and subfigure (b) illustrates the rotational frequency curve.

To validate how well the proposed method performs, we applied the LEOADRT method to analyze the signal’s time–frequency characteristics. Additionally, to comprehensively assess method performance, we conducted a comparative study with six other mainstream TFA methods, including STFT, SET, VSLCT, SBCT, GLCT, and EMCT. These comparative experiments are conducted to demonstrate the advantages of LEOADRT in handling intricate vibration signals with crossing components. To ensure the reliability and consistency of the results, all methods employed the same window length of 450.

**Figure 21:**
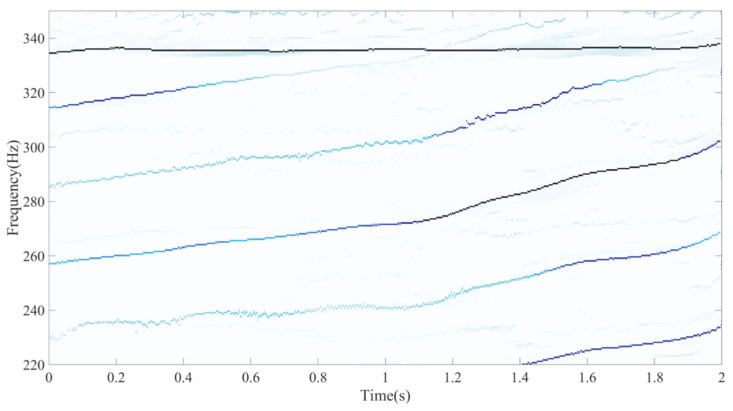
TFA results of the signal using the LEOADRT method.

**Figure 22:**
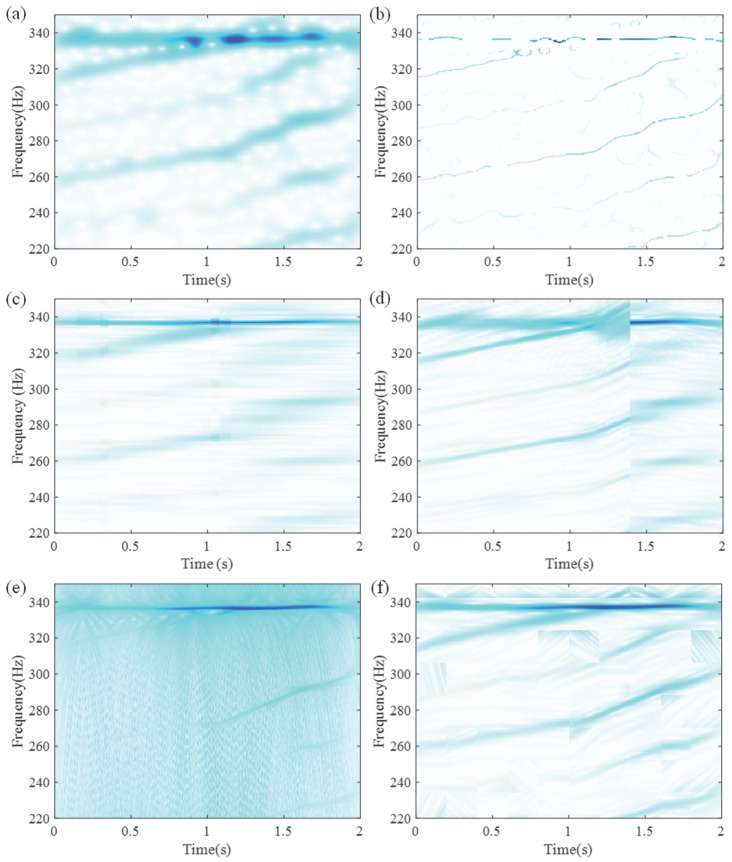
Comparison of TFA results for the signal using different TFA methods: (**a**) STFT, (**b**) SET, (**c**) VSLCT, (**d**) SBCT, (**e**) GLCT, and (**f**) EMCT.

In Figure 21, the TFA outcome produced by the LEOADRT method is presented to demonstrate its effectiveness. Figure 22a–f show the corresponding TFA results for the same signal using six other methods. From Figure 21, it is clear that some harmonic components of the turbine system can be identified, and at around 340 Hz, two IF components are observed to cross, demonstrating the method’s exceptional capability in handling crossing signals. In contrast, as shown in the results in Figure 22a–f, GLCT and VSLCT are limited to identifying only one frequency component and fail to effectively capture the low-frequency harmonics. The SET, SBCT, and EMCT methods can partially display the low-frequency harmonic components but exhibit significant scattering in the crossing regions, with unclear IF ridges and lower resolution.

To assess how well each algorithm concentrates energy in the TFR, Rényi entropy is employed as a quantitative metric. Entropy results from multiple approaches are summarized in Table 2. From the data, we find that GLCT and STFT have higher entropy values, indicating more dispersed energy in their TFR. In contrast, the proposed method has a lower Rényi entropy than SBCT, EMCT, and VSLCT, with the lowest value among them. These results demonstrate that our method attains superior energy concentration within its TFR, indicating its effectiveness in analyzing real signals with cross-characteristics.

### 5.3. The Bearing Inner Race Fault Signal Analysis Experiment

**Figure 23:**
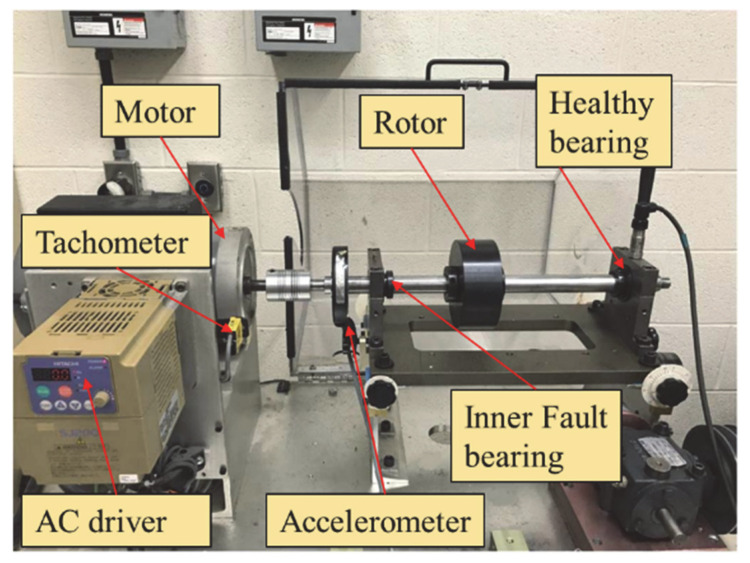
Experimental setup for bearing fault simulation.

The signal used in this experiment is sourced from a mechanical fault testing platform specifically designed for bearing fault simulation. The experimental setup, shown in Figure 23, consists of several key components, including an AC drive, motor, coupling, encoder, and bearings. During the experiment, the AC drive controls the motor speed, which drives the rotation of the shaft. The platform is equipped with two bearings located on either side, with the left bearing serving as the faulty bearing and the right bearing as the healthy reference bearing. In this experiment, the defect is located on the bearing’s inner race [39]. To monitor the bearing’s operational condition and collect experimental data, the left bearing’s outer housing is fitted with an accelerometer. By precisely controlling the fault region of the bearing, the experiment is able to simulate various fault scenarios, providing valuable data for subsequent fault diagnosis research.

**Figure 24:**
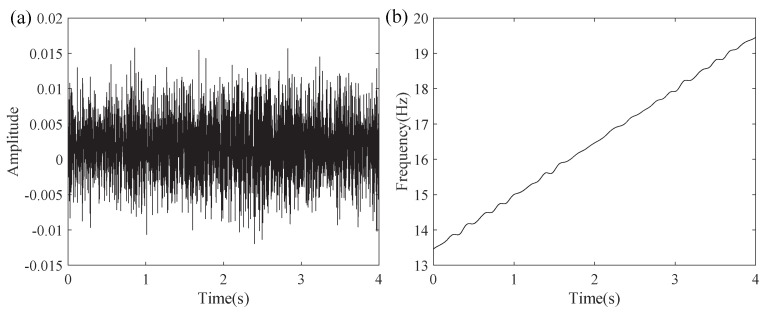
(**a**) Faulty bearing signal waveform and (**b**) speed variation over time.

In this experiment, the rotation rate of the bearing was gradually raised between 13.5 Hz and 19.5 Hz. Data acquisition used an accelerometer sampling at 200 kHz, with a 4 s analysis window. The signal was downsampled to a final sampling rate of 600 Hz, with a window length of 450. Figure 24a,b display the waveform and the speed variation over time, clearly reflecting the signal characteristics throughout the experiment.

**Figure 25:**
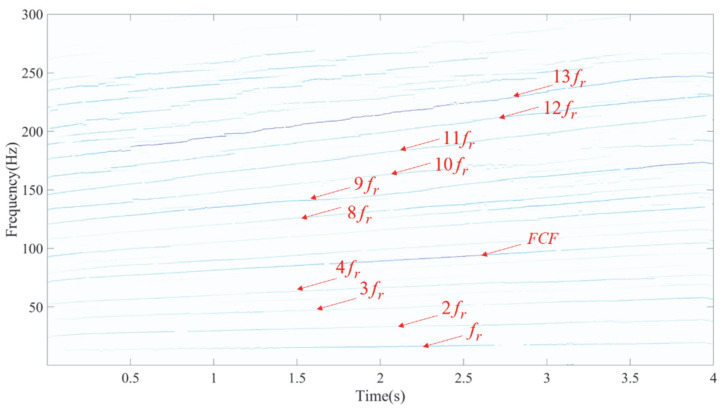
TFR of inner race bearing fault analyzed using the LEOADRT method.

Figure 25 displays the initial TFA of the signal using LEOADRT. This method’s TFR distinctly reveals the characteristic fault frequency along with its harmonics, with concentrated energy at each harmonic and superior time–frequency resolution, effectively capturing the key features of the signal.

**Figure 26:**
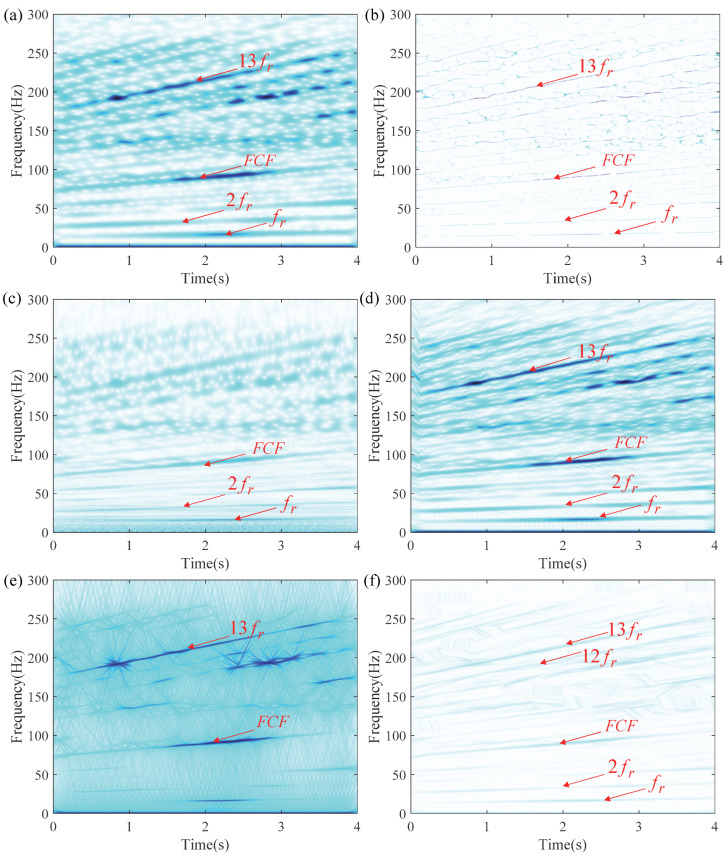
TFRs of inner race bearing fault obtained via different techniques: (**a**) STFT, (**b**) SET, (**c**) VSLCT, (**d**) SBCT, (**e**) GLCT, and (**f**) EMCT.

To conduct a more detailed comparison among TFA methods, six widely used TFA methods were applied to the same bearing signal. Figure 26a–f illustrate the analysis outcomes for each method.

By comparing the TFR generated by the six TFA methods, we observe that these methods have limitations in identifying harmonic components. Specifically, methods like STFT, SBCT, and EMCT are only able to detect lower-frequency components, such as the fundamental frequency *f_r_* and its second harmonic 2*f_r_*, with relatively low energy and poor time–frequency resolution, unable to detect the signal’s higher-order harmonic components. Additionally, these methods exhibit varying degrees of energy dispersion.

**Figure 27:**
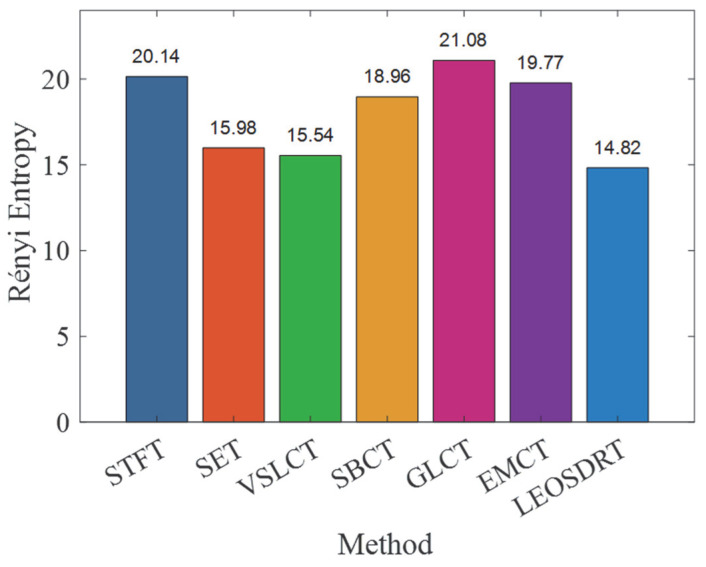
Rényi entropy comparison of 7 TFA methods.

For an extended quantitative investigation into the energy focus of the proposed method in comparison to the other six methods, we computed the Rényi entropy for each method, with the results presented in Figure 27. The figure clearly shows that the LEOADRT method yields the smallest entropy, demonstrating greater energy localization within the TFR. In contrast, the GLCT method exhibits the largest entropy, reflecting significant energy dispersion, which is consistent with the phenomena observed in its corresponding TFR.

To further evaluate the anti-interference capability and robustness of the proposed LEOADRT method, as well as six comparative methods under noisy environments, a noise-added experiment was designed in this study. Specifically, zero-mean Gaussian white noise was added to the original signals, with the SNR ranging from 0 dB to 20 dB in increments of 2 dB, resulting in 11 datasets with varying noise intensities. Under each SNR condition, all methods were applied to the noisy signals, and the robustness of their corresponding TFRs was assessed.

**Figure 28:**
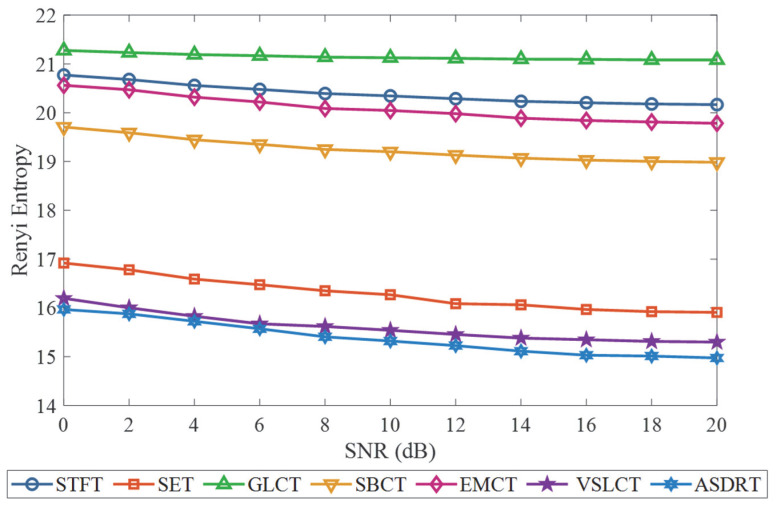
Comparison of Rényi entropy for different methods under various SNR levels.

The Rényi entropy was adopted as the evaluation metric to quantify the time–frequency concentration under different noise levels. A lower entropy value indicates that the method maintains a more concentrated energy distribution despite the presence of noise, thereby demonstrating stronger noise robustness. The variations in Rényi entropy for each method across different SNRs are illustrated in Figure 28. It can be observed that the LEOADRT method consistently yields lower entropy values across the entire SNR range, indicating superior noise resistance and time–frequency stability. These results clearly demonstrate that LEOADRT exhibits excellent time–frequency focusing capability and anti-interference performance even under high-noise conditions, highlighting its strong potential for practical engineering applications.

In conclusion, the LEOADRT method demonstrates a clear advantage in analyzing complex, non-stationary signals, particularly in the case of inner race bearing faults. Its superior energy concentration and time–frequency resolution make it highly effective for processing such signals, providing a reliable tool for subsequent fault diagnosis and prediction.

### 5.4. Future Directions

Although the proposed LEOADRT method demonstrates strong performance in enhancing the energy concentration of TFRs for non-stationary signal analysis, its current design primarily focuses on improving the accuracy of time–frequency depiction without deeply integrating intelligent algorithms. In future research, incorporating LEOADRT with advanced machine learning or deep learning techniques may provide more efficient and intelligent solutions for mechanical fault diagnosis.

On the one hand, recent developments in deep learning-based post-processing approaches for TFRs—such as CTNet and TFANet—have shown excellent capabilities in feature extraction and discrimination under complex conditions. The high-resolution and energy-concentrated TFRs produced by LEOADRT can serve as effective inputs to these networks, thereby enhancing their ability to perceive critical fault characteristics and improving diagnostic robustness. On the other hand, integrating LEOADRT with adaptive signal processing strategies—such as dynamic thresholding or segment-wise reweighting—could further enhance its adaptability to varying operational conditions and low signal-to-noise ratio environments.

Moreover, LEOADRT can be employed as a front-end feature enhancement module in combination with established deep neural network-based fault classification models, including CNNs, transformers, and GNNs. This integration enables the construction of end-to-end intelligent diagnostic frameworks that optimize the entire pipeline from signal preprocessing to fault identification. Such hierarchical integration is expected to significantly improve fault recognition accuracy, generalization across operating conditions, and real-time diagnostic performance.

## 6. Conclusions

This study introduces an innovative TFA approach, LEOADRT, designed to handle complex non-stationary mechanical vibration signals with multiple IFs or closely spaced components. The method introduces a demodulation term to compensate for CR, achieving component stabilization, and employs a local optimal theory grounded in Rényi entropy for selecting the best demodulation parameters. Energy concentration across the TFR is then redistributed based on the maximum local energy criterion, resulting in a more precise time–frequency distribution. Multiple sets of simulated and experimental signals demonstrate that LEOADRT significantly outperforms mainstream TFA methods such as SBCT, EMCT, VSLCT, and GLCT, offering superior time–frequency resolution and more concentrated energy distribution when processing non-proportional and closely spaced complex non-stationary signals.

Considering the demonstrated capabilities of LEOADRT in analyzing complex non-stationary signals, the method shows potential for application in practical industrial scenarios. For example, in mechanical systems featuring structural complexity, variable operating conditions, and moderate noise interference—such as wind power equipment, high-speed rail systems, and intelligent manufacturing platforms—LEOADRT may serve as a supportive time–frequency analysis tool, contributing to more reliable feature extraction for equipment condition monitoring and fault trend analysis.

## Figures and Tables

**Table 1:** Comparison of the computation time for different methods.

Method	STFT	SET	VSLCT	SBCT	GLCT	EMCT	LEOADRT
Computation Time (s)	0.3667	1.0961	6.6159	5.5231	12.7139	7.0982	5.9024

**Table 2:** Rényi entropy evaluation of various methods.

Method	STFT	SET	VSLCT	SBCT	GLCT	EMCT	LEOADRT
Rényi entropy	17.1873	13.4730	19.1801	15.7607	16.8372	14.9527	12.2943

## Data Availability

The data supporting the findings of this study are available from the authors upon reasonable request.

## References

[1] Jia S., Wang J., Zhang X., Han B. (2021). A weighted subdomain adaptation network for partial transfer fault diagnosis of rotating machinery. Entropy.

[2] Yan X., She D., Xu Y., Jia M. (2021). Application of Generalized Composite Multiscale Lempel–Ziv Complexity in Identifying Wind Turbine Gearbox Faults. Entropy.

[3] Tang M., Liao Y., Luo F., Li X. (2022). A novel method for fault diagnosis of rotating machinery. Entropy.

[4] Qian Q., Zhang B., Li C., Mao Y., Qin Y. (2024). Federated transfer learning for machinery fault diagnosis: A comprehensive review of technique and application. Mech. Syst. Signal Process..

[5] Zhi S., Su K., Yu J., Li X., Shen H. (2025). An unsupervised transfer learning bearing fault diagnosis method based on multi-channel calibrated Transformer with shiftable window. Struct. Health Monit..

[6] Zhi S., Wu H., Shen H., Wang T., Fu H. (2024). Entropy-aided meshing-order modulation analysis for wind turbine planetary gear weak fault detection under variable rotational speed. Entropy.

[7] Zhang J., Kong Y., Chen Z., Han T., Han Q., Dong M., Chu F. (2024). CBAM-CRLSGAN: A novel fault diagnosis method for planetary transmission systems under small samples scenarios. Measurement.

[8] Portnoff M. (1980). Time-frequency representation of digital signals and systems based on short-time Fourier analysis. IEEE Trans. Acoust. Speech Signal Process.

[9] Daubechies I. (1990). The wavelet transform, time-frequency localization and signal analysis. IEEE Trans. Inf. Theory..

[10] Boashash B., Black P. (1987). An efficient real-time implementation of the Wigner-Ville distribution. IEEE Trans. Acoust. Speech Signal Process..

[11] Flandrin P., Rioul O. Affine smoothing of the Wigner-Ville distribution. Proceedings of the International Conference on Acoustics, Speech, and Signal Processing.

[12] Rilling G., Flandrin P., Goncalves P. On empirical mode decomposition and its algorithms. Proceedings of the IEEE-EURASIP Workshop on Nonlinear Signal and Image Processing (NSIP-03).

[13] Peng Z.K., Tse P.W., Chu F.L. (2005). An improved Hilbert–Huang transform and its application in vibration signal analysis. J. Sound. Vib..

[14] Czerwinski R.N., Jones D.L. (1997). Adaptive short-time Fourier analysis. IEEE Signal Process. Lett..

[15] Auger F., Flandrin P. (1995). Improving the readability of time-frequency and time-scale representations by the reassignment method. IEEE Trans. Signal Process..

[16] Oberlin T., Meignen S., Perrier V. The Fourier-based synchrosqueezing transform. Proceedings of the 2014 IEEE International Conference on Acoustics, Speech and Signal Processing (ICASSP).

[17] Li M.F., Wang T.Y., Kong Y., Chu F.L. (2022). Synchro-reassigning transform for instantaneous frequency estimation and signal reconstruction. IEEE Trans. Ind. Electron..

[18] Yu G., Yu M., Xu C. (2017). Synchroextracting transform. IEEE Trans. Ind. Electron..

[19] Daubechies I., Lu J., Wu H.T. (2011). Synchrosqueezed wavelet transforms: An empirical mode decomposition-like tool. Appl. Comput. Harmon. Anal..

[20] Oberlin T., Meignen S., Perrier V. (2015). Second-order synchrosqueezing transform or invertible reassignment? Towards ideal time-frequency representations. IEEE Trans. Signal Process..

[21] Pham D.-H., Meignen S. (2017). High-order synchrosqueezing transform for multicomponent signals analysis—With an application to gravitational-wave signal. IEEE Trans. Signal Process..

[22] Yu G., Wang Z., Zhao P. (2019). Multisynchrosqueezing transform. IEEE Trans. Ind. Electron..

[23] Yu G., Lin T., Wang Z., Li Y. (2021). Time-reassigned multisynchrosqueezing transform for bearing fault diagnosis of rotating machinery. IEEE Trans. Ind. Electron..

[24] Mann S., Haykin S. (1995). The chirplet transform: Physical considerations. IEEE Trans. Signal Process..

[25] Czarnecki K., Fourer D., Auger F., Rojewski M. (2018). A fast time-frequency multi-window analysis using a tuning directional kernel. Signal Process..

[26] Peng Z.K., Meng G., Chu F.L., Lang Z.Q., Zhang W.M., Yang Y. (2011). Polynomial chirplet transform with application to instantaneous frequency estimation. IEEE Trans. Instrum. Meas..

[27] Yu G., Zhou Y. (2016). General linear chirplet transform. Mech. Syst. Signal Process..

[28] Miao Y., Sun H., Qi J. (2018). Synchro-compensating chirplet transform. IEEE Signal Process. Lett..

[29] Guan Y., Liang M., Necsulescu D.S. (2018). Velocity synchronous linear chirplet transform. IEEE Trans. Ind. Electron..

[30] Abratkiewicz K. (2020). Double-adaptive chirplet transform for radar signature extraction. IET Radar Sonar Navig..

[31] Li M.F., Wang T.Y., Chu F.L. (2021). Component matching chirplet transform via frequency-dependent chirp rate for wind turbine planetary gearbox fault diagnostics under variable speed condition. Mech. Syst. Signal Process..

[32] Lv Y., Ma Y., Yuan R. (2022). Velocity synchronous chirplet extracting transform: An effective tool for fault diagnosis of variable-speed rotational machinery. IEEE Sens. J..

[33] Guan Y., Feng Z. (2022). Adaptive linear chirplet transform for analyzing signals with crossing frequency trajectories. IEEE Trans. Ind. Electron..

[34] He Y., Hu M., Jiang Z., Feng K., Ming X. (2022). Local maximum synchrosqueezes from entropy matching chirplet transform. Mech. Syst. Signal Process.

[35] Wu T., Zhang W., Zhang B., Luo H. (2023). Application of multi-base fusion generalized chirplet basis transform in vibration signal analysis of multiple rotor rotating machinery. Mech. Syst. Signal Process..

[36] Zhao D., Wang H., Huang X., Cui L. (2024). Local optimal scaling chirplet transform for processing nonstationary mechanical vibration signals. IEEE Trans. Instrum. Meas..

[37] Miaofen L., Youmin L., Tianyang W., Fulei C., Zhike P. (2023). Adaptive synchronous demodulation transform with application to analyzing multicomponent signals for machinery fault diagnostics. Mech. Syst. Signal Process..

[38] Wu H., Zhi S., Fang Q., Liu Y., Wang T., Cheng W., Chu F. (2024). Synchronous decomposition match-reassigning transform and its application in planetary gearbox fault diagnosis. Meas. Sci. Technol..

[39] Huang H., Baddour N. (2018). Bearing vibration data collected under time-varying rotational speed conditions. Data Brief.

